# Ultrafast Charge Carrier Dynamics in Vanadium Dioxide, VO_2_: Nonequilibrium Contributions to the Photoinduced Phase Transitions

**DOI:** 10.1021/acs.jpclett.4c02951

**Published:** 2025-01-28

**Authors:** John A. Tomko, Kiumars Aryana, Yifan Wu, Guoqing Zhou, Qiyan Zhang, Pat Wongwiset, Virginia Wheeler, Oleg V. Prezhdo, Patrick E. Hopkins

**Affiliations:** † Department of Mechanical and Aerospace Engineering, University of Virginia, Charlottesville, Virginia 22904, United States; ‡ Department of Chemistry, University of Southern California, Los Angeles, California 90089, United States; § Department of Physics and Astronomy, University of Southern California, Los Angeles, California 90089, United States; ∥ U.S. Naval Research Laboratory, Washington, D.C. 20375, United States; ⊥ Department of Materials Science and Engineering, University of Virginia, Charlottesville, Virginia 22904, United States; # Department of Physics, University of Virginia, Charlottesville, Virginia 22904, United States

## Abstract

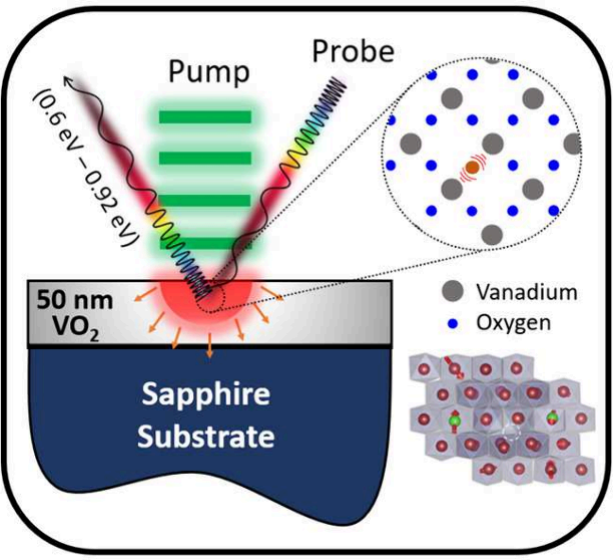

Vanadium oxide (VO_2_) is an exotic phase-change material with diverse applications ranging from thermochromic smart windows to thermal sensors, neuromorphic computing, and tunable metasurfaces. Nonetheless, the mechanism responsible for its metal–insulator phase transition remains a subject of vigorous debate. Here, we investigate the ultrafast dynamics of the photoinduced phase transition in VO_2_ under low perturbation conditions. By experimentally examining carrier relaxation dynamics at energy levels near the VO_2_ band gap (0.6–0.92 eV), we note that numerous optical features do not correspond to the first-order phase transition. Previous studies indeed induced such a phase transition, but they relied on fluences at least an order of magnitude higher, leading to temperature increases well above the transition threshold (340 K). Instead, for excitation fluences that correspond to lattice temperatures only in slight excess of the phase transition (absolute temperatures < 500 K), we find that the marked changes in optical properties are dominated by a shift in the electronic density of states/Fermi level. We find that this effect is a lattice-driven process and does not occur until sufficient energy has been transferred from the excited electrons into the phonon subsystem.

Vanadium dioxide (VO_2_) is the quintessential electron-correlated material, having served as the testbed for understanding metal–insulator transitions (MIT) in metal oxides for decades; a number of reviews have summarized the countless studies that have investigated the corresponding mechanism for this phase transition in VO_2_.^1−5^ These efforts are driven by not only the technological relevance of MITs, due to the marked change in thermal,^6,7^ optical,^8−10^ and electrical^6,11,12^ properties that occur across the transition temperature, but also by the fact that VO_2_’s MIT occurs just above room temperature (∼340 K), thus increasing experimental accessibility. While many of the reviewed works have greatly increased the technological applicability of VO_2_ by manipulating either the transition temperature,^13,14^ the degree of property manipulation,^15^ or the speed of modulation,^16^ all of which can be considered metrics of performance, fewer studies have been devoted to understanding the fundamental mechanism of this MIT. To date, these investigations into the fundamental nature of the MIT have relied on two primary frameworks, typically striving to answer one explicit question: VO_2_: Peierls or Mott-Hubbard?^17^ In other words, is the phase transition driven by changes in the atomistic structure and induced by lattice instabilities as proposed within the Peierls framework,^18,19^ or is it a Mott-type transition, where electron correlations lead to a collapse of the band gap?^20^ Although these competing theories were initially proposed as a way of explaining the observed modulation of electronic properties upon doping of transition-metal oxides, the advent of ultrafast metrologies has led to the application of these formalisms to more sophisticated studies that are capable of directly interrogating electron^21−24^ and lattice^25−28^ dynamics. Despite such advances in both experimental and computational methods, there remain conflicting reports suggesting that the mechanism could be Peierls, Mott, a combination of the two, or ill-described by either;^29,30^ a unified understanding remains elusive.

A common approach to identifying the mechanism of the MIT in time-resolved spectroscopy methods is simply evaluating the time scale over which the electrons or lattice undergoes significant changes. For example, if a significant increase in electrical conductivity is observed following optical pulse heating, before electron–phonon scattering collisions occur, it strongly suggests that the MIT is not driven by lattice instabilities. In such a scenario, the population of the phononic subsystem would not yet have been modulated on the time scale associated with the observed MIT. Indeed, this logic has been applied to experimental reports on both Peierls/lattice^28,31,32^ and Mott/electronic^21,31,33−36^ transitions through ultrafast pump–probe measurements with a variety of spectroscopic techniques, ranging from ultrafast X-ray diffraction measurements to terahertz (THz) frequency probes.

A majority of the ultrafast pump–probe studies have thus far relied on high-energy, low-repetition-rate laser systems to induce and interrogate the MIT. The general consensus is that no energy remains from previous laser pulses, which would convolute the results or induce material degradation. Thus, each pulse must individually have sufficient fluence to exceed the transition temperature thermally or an electric field in excess of that necessary to strongly perturb the electronic subsystem. While these studies are certainly of merit, we hypothesize that a large perturbation of the system with high fluence may introduce its own complications. In fact, a recent theoretical work by Liu et al.^37^ has shown the strong correlation between the number of photoexcited carriers with the order or disorder phase transition in VO_2_. Similarly, a photoinduced metallic metastable phase arising from local atomic nucleation has been postulated very recently to explain the experimental data for the photosusceptibility of VO_2_^38^ and time-resolved harmonic spectroscopy in NbO_2_.^39^

First, in transient reflectivity/transmission experiments, a majority of thermo-optical models rely on an assumption that the system is within a low-perturbation limit, such that the response function remains linear with respect to the variable of interest (e.g., electron or lattice temperatures).^40^ However, in experiments utilizing single, high-energy pump pulses, the system inherently is in a state of strong nonequilibrium, which has been shown to induce nonlinearities in the optical response of materials, even in the case of gold,^41^ which has been one of the more commonly studied material systems for understanding electron–phonon nonequilibrium in time-resolved studies via these thermo-optical relationships. Second, these states of strong nonequilibrium lead to a deviation of thermophysical properties that are necessary for interpretation and deconvolution of the electron and phonon subsystems. For example, one may naively assume that switching speeds of ≲100 fs correspond to a temporal regime prior to lattice heating, as the electrons do not have sufficient time to couple to the phononic subsystem. Yet at sufficiently high pulse energies, this assumption no longer holds true, and the lattice can be “instantaneously” heated to temperatures in excess of the MIT threshold due to an increased electron–phonon coupling rate.^42^ Finally, many studies have relied on probing at energies corresponding to wavelengths in the visible spectrum as most commercial laser systems operate in this region. Yet the band structure of interest lies at much lower energy levels, corresponding to infrared wavelengths (*E*_G_ ≈ 0.7 eV), where analysis may be pertinent toward understanding the MIT mechanism.

We note that an equally large number of works have relied on ultrafast optical pump X-ray probe,^25,28,35^ deep-UV probe,^43^ photoelectron probe,^21^ and electron-scattering probe^32^ measurements, of which interpretation is less convoluted by these issues; a thermo-optical model is less necessary, as these methods directly interrogate the structure of interest. While these works are certainly of merit, they rely on excessively high excitation events and orders of magnitude larger pump fluences than typically observed as “necessary” for inducing the phase transition in VO_2_ when compared to optical measurements. In doing so, it is very possible that the transition dynamics are drastically different from those of a near-equilibrium setting.

In this work, we perform a series of ultrafast measurements specifically designed to trigger MIT in VO_2_ by lightly perturbing the material with low-energy optical pulses. These pulses provide sufficient energy to slightly elevate the lattice temperature by only a few Kelvin. Further details on the experimental setup are provided in Supporting 1. To ensure that the residual temperature rise from earlier pulses persists until the next pulse reaches the sample, we utilize a high-repetition-rate oscillator operating at 80 MHz (Figure 1a,b). As a result, the lattice no longer remains at room temperature during measurement but maintains a steady-state temperature slightly below the phase-transition temperature (340 K). This controlled approach ensures that the lattice stays a few Kelvin below the MIT temperature, allowing the optical pulses to provide just enough energy to trigger the phase transition without inducing strong nonlinearities in the system. Second, we interrogate VO_2_ at wavelengths spanning the near-infrared spectrum (1350–2050 nm or ∼0.92–0.60 eV) to investigate potential narrowing of the band gap during the photoinduced transition. As described in this work, we find that signatures commonly attributed to the photoinduced transition of VO_2_ do not occur until the electrons have coupled sufficient energy to the phononic subsystem, indicating a lattice-driven mechanism in this low-perturbation limit. However, by extending these measurements toward *insitu* monitoring of the band gap following ultrafast excitation of the VO_2_, we observe no discernible collapse of the band structure for the fluences used in this work, despite similar signatures that have been previously attributed to the observation of a photoinduced phase transition. In other words, our results indicate that, for at least the fluences investigated in this work, large changes in the modulated optical spectrum of phase-change materials are not intrinsically related to the MIT but can instead be indicative of ultrafast band renormalization due to atomic (e.g., defect) oscillations. These results have strong implications on the time scale and rate at which MIT can be accessed and manipulated.

**Figure 1:**
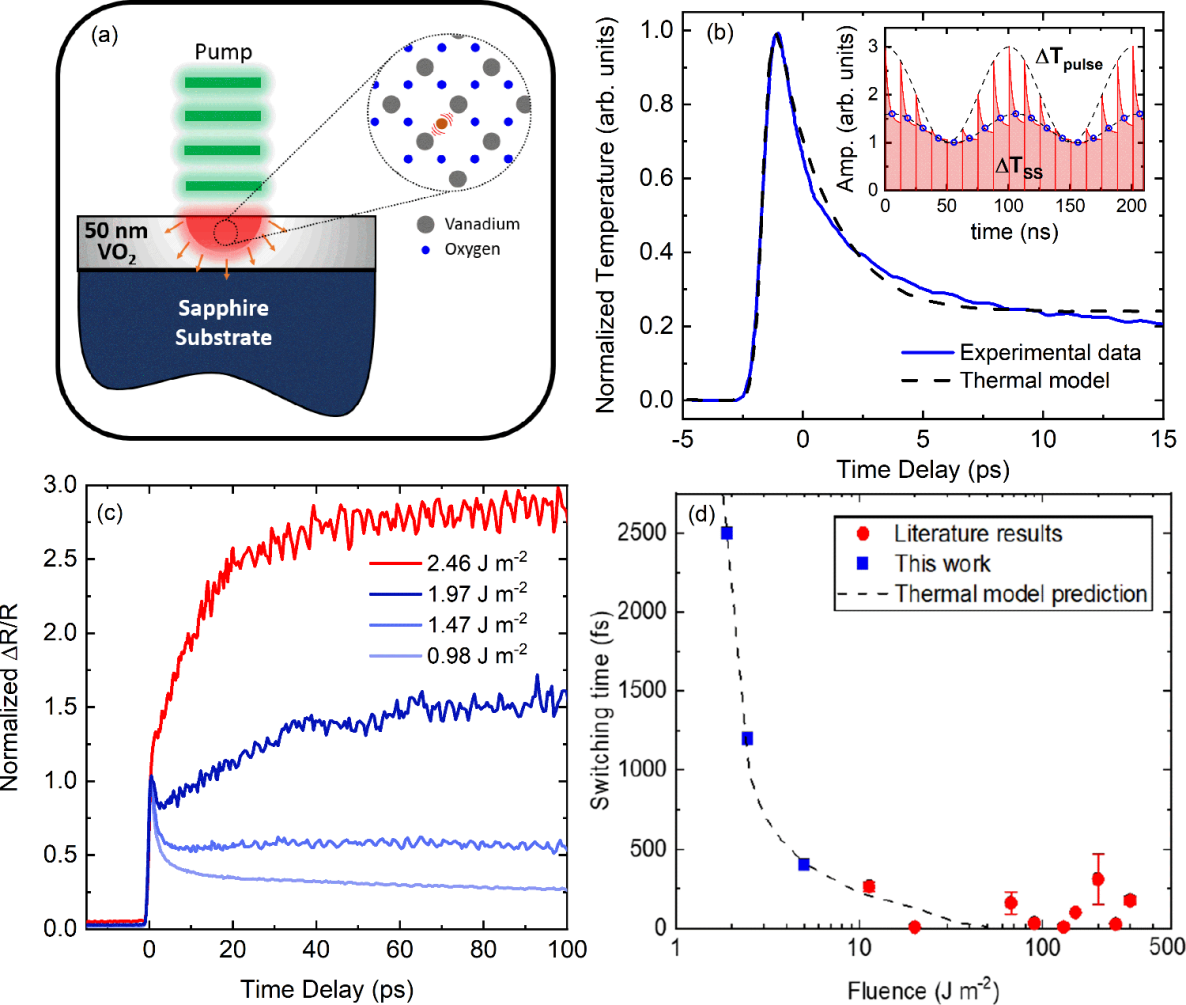
a) Schematic of the measurement technique and the VO_2_ with defects. b) Two-temperature model (TTM) fit (dashed black line) to our experimental transient reflectivity measurements (blue line), providing a detailed understanding of the energy transfer rates between electrons and phonons within VO_2_. The inset shows the periodic temperature rise due to ultrafast pump–probe experiments and their corresponding exponential thermal decay. At high modulation frequencies, as used in this work, there are two contributions: a relatively constant “background” steady-state temperature rise (Δ*T*_*SS*_) due to pulse accumulation and the more often considered “impulse” temperature rise due to individual pulses (Δ*T*_*pulsed*_). c) Transient reflectivity data measured at 800 nm for varying pump fluences. There is a marked change in dynamics beginning at 1.5 J m^–2^; this change has been previously associated with a photoinduced phase transition.^23,32,33,43−45^ d) Predicted switching time for a lattice-driven phase transition (i.e., how long before the electrons couple sufficient energy to the lattice to exceed 340 K) compared to our experimental results and a number of reported transition times reported in the literature.^21,32,34,43,46−48^

In the case of our high-repetition-rate measurements, there are two primary “temperature rises” within the VO_2_ system: one due to the accumulation of energy between multiple pump pulses (Δ*T*_*SS*_) and the commonly investigated single-pulse excitation of the system (Δ*T*_*pulsed*_). These periodic temperature increases are graphically depicted in the inset of Figure 1b. Here, we rely on the combination of these effects to require only small per-pulse perturbations to the system (e.g., minimize the contribution of Δ*T*_*pulsed*_) to induce an observed photoinduced phase transition. For example, in our experiment, at a fluence of ∼1.5 J m^–2^, Δ*T*_*SS*_ contributes a steady-state temperature rise of nearly 40 K, requiring Δ*T*_*pulsed*_ to be on the order of only a few Kelvin to exceed the phase-transition temperature of VO_2_. This is in contrast to previous works that rely on the impulse temperature rise, Δ*T*_*pulsed*_, to be in excess of 45 K for such an event to occur.

A series of ultrafast transient reflectivity measurements are performed at varying fluences utilizing this high-repetition-rate oscillator, with a summary of the results shown in Figure 1c. There are a few salient features to note. First, at low fluences, the transient reflectivity is qualitatively similar to that of most metals, where there is an initially large peak due to the rapid increase in electron temperature from photoexcitation; this peak decays over a few picoseconds via energy transfer from the electrons to the lattice of the material (e.g., electron–phonon coupling). These processes are followed by a decay in the measured signal as energy flows away from the heated surface into the bulk of the system through phonon-dominated conduction. More interestingly, we observe that with increasing fluence the latter portion of these dynamics begins to change. As shown in Figure 1c, at fluences exceeding ∼1.5 J m^–2^, the transient reflectivity begins to show a *second* increase in the signal; the onset of this increase occurs at earlier times as the fluence increases. Note that the electronic excitation and subsequent coupling to the lattice show no observable change prior to the occurrence of this secondary rerise. With increasing fluence (e.g., a fluence of ∼2.5 J m^–2^), this secondary rerise becomes significant, beginning at a pulse-width-limited time scale of 400 fs, and the transient reflectance increases by nearly 300%. This observation is again in agreement with prior works and has been associated with the photoinduced phase transition of VO_2_.^23,32,33,43−45^ We note that at fluences of less than ∼1 J m^–2^, the temporal dynamics are identical and mimic that of the lowest fluence shown in Figure 1c.

To gain initial insight into the mechanism driving this rerise in our transient reflectivity data, we perform two-temperature model (TTM) calculations.^49^ The TTM allows us to understand energy transfer between the electron and phonon subsystems, thus determining which process dominates the marked change in ultrafast optical properties of VO_2_ through the solution of the following two equations,
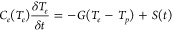
1
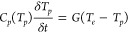
2where *C* and *T* are the heat capacity and temperature of the respective subsystems, *G* is the electron–phonon coupling factor of the material, and *S* is the laser source term. It is critical to note that the application of the TTM relies on our aforementioned assumption of remaining in a “low-perturbation limit” of transient reflectivity, as the relationship between temperature and reflectivity quickly becomes nonlinear for large energy excursions. Additionally, within this limit, *G* and other thermophysical parameters can be considered to be constant; for large temperature excursions, the energy transfer rate between electrons and phonons can vary by orders of magnitude from its room-temperature value.^42^ An example fit of the TTM to our experimental data, as a means of extracting *G* and the thermo-optic coefficients, is shown in Figure 1b. With the thermophysical properties of VO_2_ at hand, we can first consider the case of a lattice-driven process: how long will it take for the phonons to have sufficient energy to exceed the phase-transition temperature of VO_2_ following optical excitation? The resulting time for the system to exceed ∼340 K based on these calculations is shown in Figure 1d, along with the time at which we observe a rerise in our signal as well as a comparison to the reported transition times for a number of previous reports in the literature. It is clear that the observed rise in signal aligns well with the time necessary for electrons to transfer their energy to the lattice. Thus, in this low-perturbation limit, one would believe that the photoinduced phase transition of VO_2_—the assumed origin of this rerise in transient reflectivity—can be attributed to a lattice-driven process.

To gain further insight into this observation, we extend our measurements to an ultrafast optical pump–tunable infrared probe system.^50^ By tuning the probe wavelength through the band gap of VO_2_, we can monitor the onset of band collapse during this rerise in signal; in other words, we use ultrafast time-resolved “Tauc plots” to measure the band gap following photoexcitation.^51,52^ Additionally, the phase transition of VO_2_ is commonly utilized for the modulation of its infrared properties for applications such as passive cooling, thus the infrared properties during the ultrafast, photoinduced phase transition are of particular interest to a number of technologies. Our measurements provide direct insight into the time scale and magnitude of the changes in near-infrared optical properties of VO_2_ following optical excitation. These results are summarized in Figure 2, with an example of both combined temporal and spectral data from these experiments shown in Figure 2a. An outline of the salient features of this contour is in Figure 2b–d and discussed in the following text.

**Figure 2:**
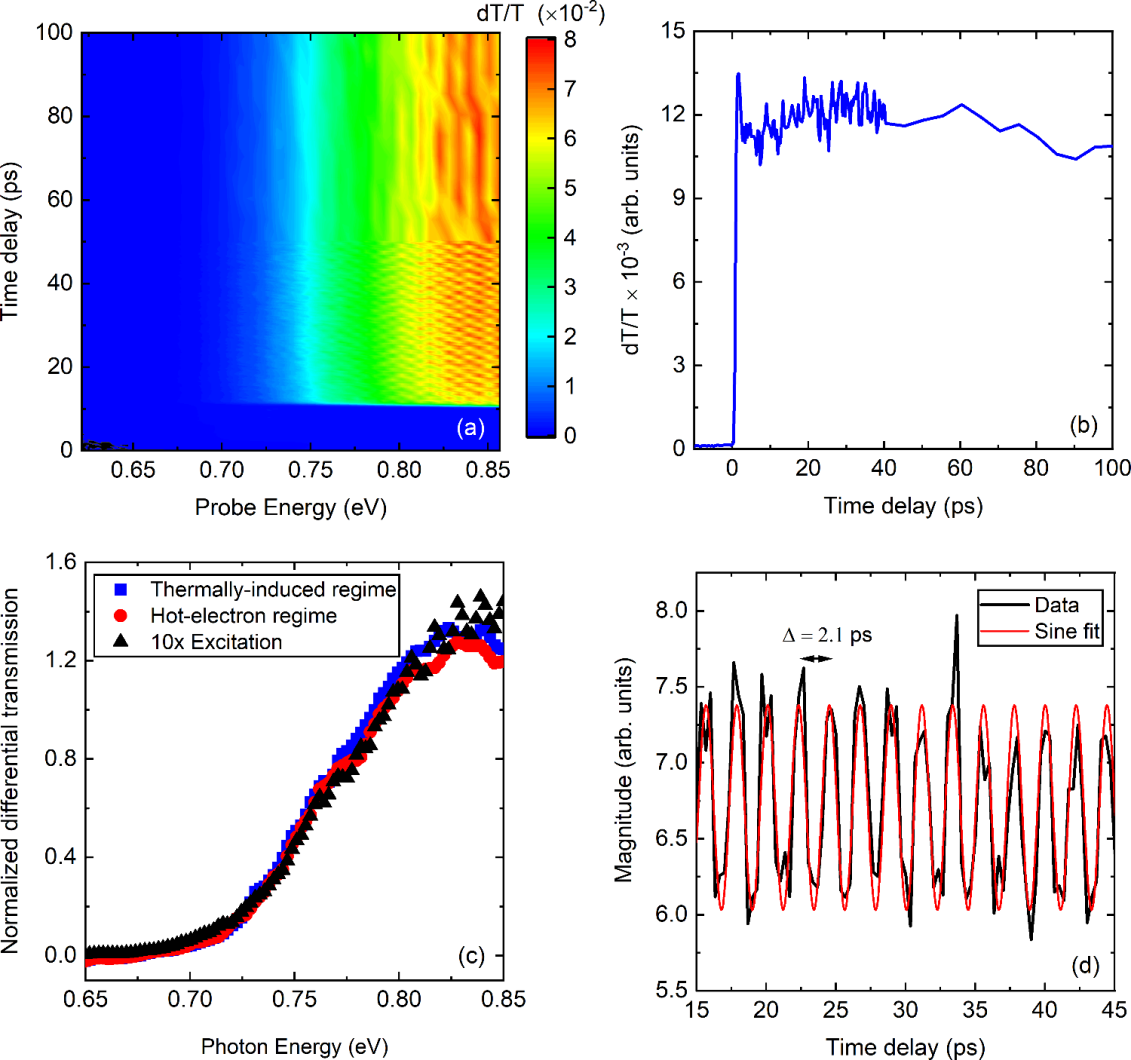
a) Contour of our transient absorption measurements with varying probe photon energies corresponding to wavelengths in the near-infrared region. b) Example cross section of the contour at one wavelength (1450 nm); at this fluence, the transient dynamics are similar to those measured in TDTR near the transition threshold (e.g., a “rerise” in the signal is observed). c) Spectral cross-section of the same contour figure immediately following excitation (i.e., zero time delay between pump and probe, red circles), where the electrons are out of equilibrium with the lattice and are at an elevated temperature, as well as at much later times where the lattice has gained sufficient energy to exceed 340 K and is in equilibrium with the electronic subsystem (blue squares). Additionally, the spectral cross-section for a fluence that is 10× greater and the “rerise” dominates the entirety of the transient absorption measurement (black triangles). In all cases, the normalized transmission shows no spectral change, indicating a lack of band collapse. d) Transient absorption magnitude as a function of time delay shows a highly periodic oscillation.

If we consider a temporal cross-section of these data (e.g., one wavelength as a function of the pump–probe time delay), we find dynamics nearly identical to those obtained with our 80 MHz, 800 nm probe measurements shown in Figure 1c, where at a fluence that corresponds to a calculated lattice temperature of 340 K we begin to see the onset of a rerise in our transient absorption measurement, deviating from the response of typical energy decay into the bulk of a material system. With increasing fluence, this rerise becomes more prominent and begins to occur at earlier times, which we assigned to the time of which the lattice temperature exceeds a critical value. These dynamics are consistent across all probe wavelengths used in this work.

More importantly, these measurements provide access to the spectral variations in transient absorption; a number of these spectral cross sections of the contour plot are shown in Figure 2c. We consider three critical regimes: when the electrons are in a state of strong nonequilibrium with respect to the lattice (*T*_*e*_ ≫ *T*_*p*_); at later times during the rerise, when the two subsystems have reached equilibrium with respect to each other (*T*_*e*_ ≈ *T*_*p*_ > 340 K); and the same scenarios at fluences much greater than the threshold of this onset, where the rerise dominates the entirety of the transient absorption signal (e.g., a regime that has been typically associated with a photoinduced phase transition; *T*_*e*_ + *T*_*p*_ ≫ 340 K). In *all* cases and for all pump–probe time delays, we find that the normalized transient absorption spectrum (the absolute magnitude of transient absorption scales with fluence) is identical: there is a marked increase in the response of the system for energies exceeding the band gap of VO_2_, but the curvature of this spectrum is neither fluence- nor time-dependent. As the spectral derivative of transient absorption is strongly dependent on the optical band structure of a material, it has commonly been used as a means of determining critical points and optically induced band transitions. In the case of a direct gap insulator such as VO_2_, one would expect the low-temperature spectrum to mimic that of a Tauc plot,^53−55^ which is indeed our observation. Interestingly, despite the “rerise” signature assigned to a photoinduced phase transition in the temporal data, these spectral derivatives indicate that there is no significant change in the optical band gap for the fluences investigated in this work. First, this observation further supports the evidence that we are truly in a low-perturbation, linear response regime of the pump-induced optical changes; the response is linear with respect to the excitation energy. Furthermore, the lack of an observed collapse in the optical band gap leads to a suspicion that this rerise signature associated with the photoinduced phase transition is perhaps not the monoclinic to tetragonal MIT of VO_2_ but instead is due to a separate mechanism.

To understand these results in more detail, we perform ab initio calculations on VO_2_ combining real-time time-dependent density functional theory (TD-DFT) and nonadiabatic molecular dynamics. This computational method has been developed specifically for understanding photoinduced dynamics in cases of strong electron–phonon nonequilibrium.^50,56−58^ Further details about the calculation parameters can be found in Supporting 2. The calculations reveal several important aspects of the photoinduced behavior of VO_2_.

With regard to the initial exponential decay following photoexcitation (e.g., the electronic relaxation dynamics prior to any rerise in transient reflectivity) (Figure 1c), our calculations show that *pristine* VO_2_ (e.g., a perfect, defect-free crystal) should have an electron–hole recombination time longer than 50 ps (Figure 3c). This value is over an order of magnitude larger than the experimental recombination time from any known literature reports. However, repeating these calculations with the inclusion of either oxygen vacancies or interstitials (with a concentration on the order of the equilibrium defect concentration in VO_2_), the recombination time is reduced to about 3 ps (Figure 3f,i), in close agreement with the experimentally observed recombination times in both our work and prior literature reports. In other words, our TD-DFT calculations reveal that an equilibrium concentration of oxygen defects strongly affects the electron–hole recombination time in VO_2_.

**Figure 3:**
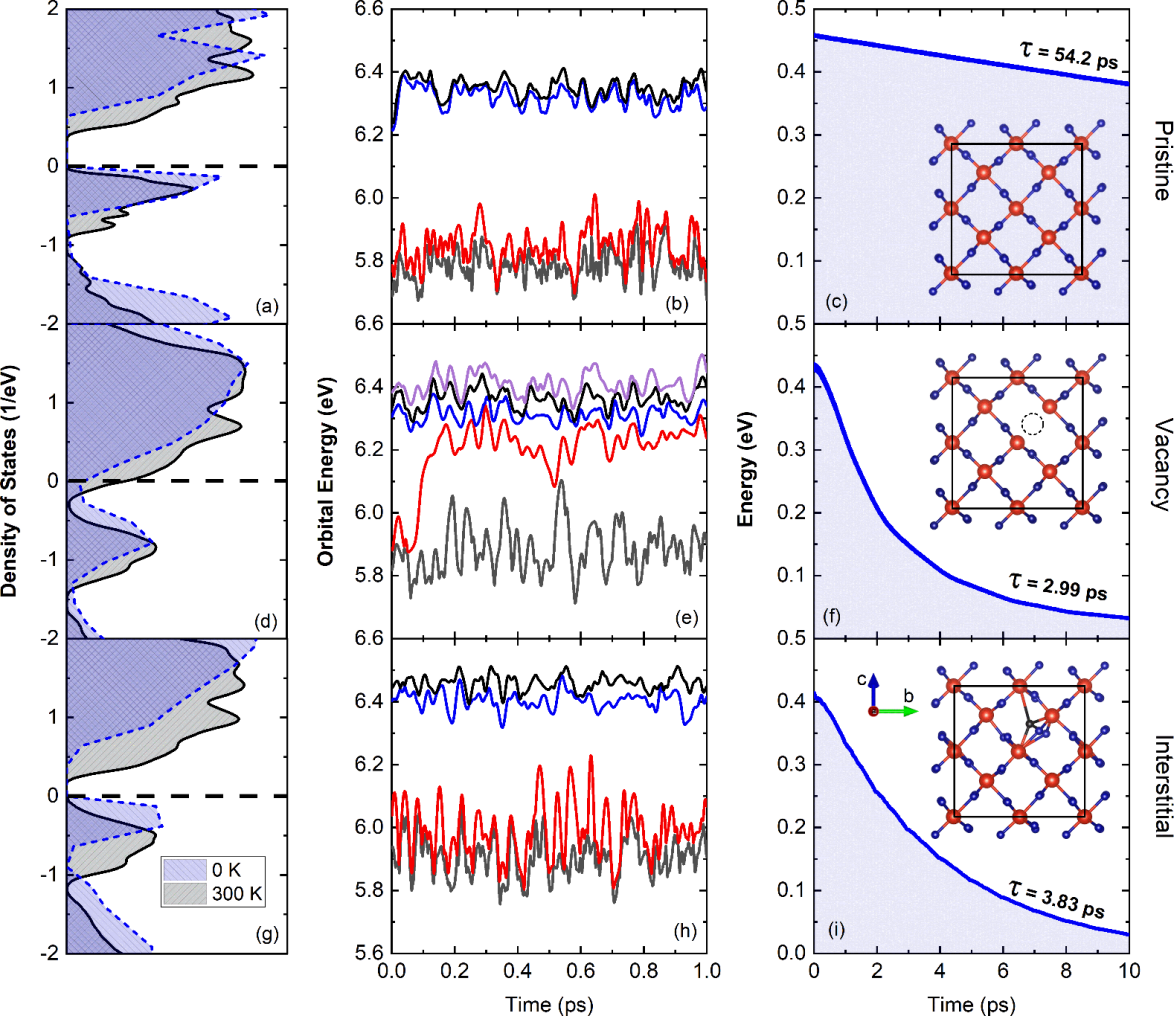
Ab initio results for (a–c) pristine VO_2_, (d–f) VO_2_ with an oxygen vacancy, and (g–i) VO_2_ with interstitial oxygen. Electronic density of states at (a, d, g) 0 K and canonically averaged at 300 K. (b, e, h) Evolution of state energies near the band gap. The electronic states and structural instabilities introduced by the defects result in large fluctuations of the fundamental band gap of VO_2_, which begins to exhibit metallic character. (c, f, i) Relaxation of excited electron’s energy resulting in electron–hole recombination. The recombination takes about 3 ps in the defective systems, in agreement with our experiments (Figure 2b,c). The insets show the front or side views of the optimized structures. The vacancy and interstitial are labeled by the dotted circles. Small red spheres and large blue spheres represent O and V atoms, respectively.

In addition to their role in electron–hole recombination, we also find that the oxygen defects are the most likely candidate for the observed “rise” in transient reflectivity in both our experiments and prior literature reports. Our TD-DFT calculations find that the transient band dynamics of photoexcited monoclinic VO_2_ are strongly affected by the presence of oxygen defects. Namely, the *abinitio* calculations demonstrate that photoexcited VO_2_ exhibits elements of metallic behavior when oxygen vacancies and interstitials are present. Oxygen vacancies create defect states near the conduction band (CB) maximum, resulting in intrinsic *n*-doping and shifting of the Fermi level to the CB (Figure 3d). The defect level fluctuates strongly across the band gap (Figure 3e), exhibiting bimodal behavior. The defect energy levels created by the oxygen interstitial reside inside the valence band (VB), but the structural instability leads to a large fluctuation of the VB maximum (VBM), transiently almost closing the band gap (Figure 3e). The fluctuations of the energy levels in the defective systems are responsible for the rapid electron–hole recombination seen in the experiment. We note that this metal-like behavior is not observed in our calculations of pristine VO_2_.

Such metal-like behavior, upon excitation to a threshold fluence, could certainly be responsible for the observed “rerise” in transient reflectivity based on several factors. First, recall our high-repetition-rate results: this ultrafast signature occurs only after the lattice has gained a sufficient amount of energy from photoexcited carriers. Similarly, the nuclear motion of oxygen defects that give rise to metallic behavior would have an energy barrier, where the fluctuation of the VBM can occur only once the defects have obtained sufficient energy. Second, neither the TD-DFT behavior nor our experimental observations indicate a collapse of the band gap, at least for the moderate fluences considered in this work, yet this significant increase in reflectivity would be observed due to the large VBM fluctuation toward the CB near the defects, creating local metallic behavior. Finally, we note a third observation in our ultrafast pump–probe data that strongly suggests that the nuclear motion of oxygen defects is responsible for this transient rise in reflectivity. As shown in Figure 2d, our ultrafast pump–probe data displays oscillatory behavior with a periodicity of approximately 2 ps; this period is far too long to be associated with the phonon dynamics of VO_2_, picosecond acoustics in the film itself, or Brillouin scattering within the sapphire substrate. However, this period is on the order of the fluctuation of the energy level that crosses the band gap in the oxygen vacancy system observed in our TD-DFT calculations (Figure 3e).

Thus, we attribute the rerise in the signal with increasing fluence/temperature during ultrafast measurements to the local shift of the VBM of VO_2_ due to the nuclear motion of oxygen defects, which can occur without a collapse of the bulk band gap and give rise to metallic behavior, despite the lack of an MIT. In other words, there remains an ∼0.7 eV energy level difference between the d_∥_ states and the π* states, while the defect levels shift into the d_∥_ DOS (Figure 3). Indeed, as the lattice gains sufficient energy from the electrons due to rapid electron–phonon relaxation around the defects, the VBM approaches the CB and begins to cross over. Thus, despite the remnant band gap in regions of pristine VO_2_, there is a rapid increase in the joint density of states, leading to a massive overall change in transient reflectivity.

Although the transient temperature rise exceeds that of the first-order phase transition associated with VO_2_, the lack of an “instantaneous” or even ultrafast change in crystal structure may not be surprising; thus far, we have taken no consideration of the kinetics of a first-order phase transition. Recall that our predicted switching time from TTM calculations considers only the amount of time necessary for sufficient energy to transfer from the heated electrons to the lattice and does not include time–temperature transformation considerations. In these low perturbation limits, where the temperature rise is on the order of the transition temperature, heat is dissipated from the sample more rapidly than the phase transition can occur. While there is sufficient experimental evidence for a photoinduced phase transition at much higher fluences than used in this work, they also correspond to much larger temperature excursions where the transformation time will be reduced.

The MD trajectories of pristine VO_2_ and VO_2_ with an oxygen vacancy and interstitial defects have indicated that the vacancy system exhibits metallic character, as shown in Figure 2. In order to investigate this further, we generated a long, 50 ps trajectory for the oxygen vacancy system, confirming large fluctuations and frequent collapse of the fundamental band gap. We analyzed the trajectory in order to establish the structural origin of this behavior. In particular, we computed the pairwise mutual information (MI)
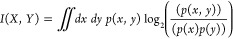
3between the band gap (*X*) and distances (*Y*) between all pairs of atoms in the system. MI allows one to identify nonlinear correlations among different properties. The data shown in Figure S3a identifies a particular pair of atoms, the distance between which correlates strongly with the band gap. The atoms are marked in green in Figure S3(b). The band gap–distance correlation revealed by the MI analysis is illustrated further by plotting the band gap and the distance in Figure S2(b–d). Importantly, the two atoms, the distance between which correlates strongly with the band gap, give rise to a 500 GHz normal mode, shown by the arrows in Figure S3(b). The 2 ps period of this mode closely matches the experimental signal shown in Figure 1(d).

In summary, in this study, we investigated the photoinduced phase transformation of VO_2_ in a low perturbation limit, where the calculated lattice temperature rise is only in slight excess of the first-order phase-transition temperature. For our optical pump–probe experiments, we find excellent agreement with the literature, where a rise in the transient reflectance was attributed to the MIT of VO_2_ (e.g., monoclinic to tetragonal crystal structure). Further investigations into the ultrafast dynamics via transient absorption measurements performed with probe wavelengths spanning the optical band gap (∼0.7 eV) indicate that the observed rerise is in fact not associated with the first-order phase transformation; while prior works have certainly induced such a phase transition, they have relied on fluences at least an order of magnitude greater and thus are inducing temperature excursions in extreme excess of ∼340 K. Instead, for excitation fluences that correspond to lattice temperatures only in slight excess of the phase transition (absolute temperatures < 500 K), we find that the marked changes in optical properties are dominated by a shift in the electronic density of states due to the nuclear motion of oxygen defects. We find that this effect is a lattice-driven process and does not occur until sufficient energy has been transferred from the excited electrons into the phonon subsystem. These results have important implications for ultrafast switching applications of VO_2_, as the system must be excited into a state of very strong nonequilibrium to induce the first-order phase transition of interest in an array of technologies.

## Data Availability

The data that support the findings of this study are available from the corresponding author upon reasonable request.

## Supporting Information Available

Details on tunable wavelength ultrafast pump–probe spectroscopy and computational methods; experimental setup for the low-perturbation pump–probe technique including the pump beam to heat the sample and a probe beam with wavelength tunability in the near IR to measure the changes in the optical properties of VO_2_; evolution of energy levels near the band gap in VO_2_ with an O vacancy over 50 ps; evolution of the band gap and the distance between the two V atoms highlighted in green in Figure S3b over the first 3 ps of the 50 ps trajectory and over 50 ps; mutual information correlating the band gap and distances between all pairs of atoms in the VO_2_ system with the O vacancy; and normal mode with 500 GHz frequency, exhibiting strong contribution from the two V atoms shown in green and having a strong correlation with the band gap (PDF)
Transparent Peer Review report available (PDF)
