# Supplementary material for: Ultrafast Charge Carrier Dynamics in Vanadium Dioxide, VO2: Nonequilibrium Contributions to the Photoinduced Phase Transitions
Source: J Phys Chem Lett. 2025 Jan 28;16(5):1312–9. doi: 10.1021/acs.jpclett.4c02951 (PMC11808786; doi:10.1021/acs.jpclett.4c02951)
Supplement: Supplementary file 1 — jz4c02951_si_001.pdf [file jz4c02951_si_001.pdf]

Supporting Information
Ultrafast charge carrier dynamics in Vanadium Dioxide, VO_2: Non-equilibrium contributions to the photo-induced phase transitions

Supporting 1 - Tunable-Wavelength Ultrafast Pump-Probe Spectroscopy

Pump-probe spectroscopy is an established measurement technique for investigating the ultrafast transport dynamics of charge carriers in materials on the order of sub-picoseconds [1,2]. In this technique, and pertinent to the results presented in this work, the output a high-power femtosecond laser (Spectra Physics 30W Spirit-HP) with pulse duration of 400 femtoseconds at the fundamental frequency of 1040 nm with repetition rate of 500 KHz is split into two portions with varying amplitude; the beam with stronger amplitude (pump) is frequency-doubled to the wavelength of 520 nm and directed to a mechanical delay stage for advancing pump pulses relative to probe in time. The pump is modulated at the frequency of 451 Hz with a chopper and then is focused the surface of the target material, VO_2, with spot size on the order of 100 um. The weaker beam (probe) is passed through an optical parametric amplifier (OPA), which filters out the *Idler* beam and operates in the *Signal* mode. This enables spectrally varying the probe wavelength from 1300 to 2000 nm.

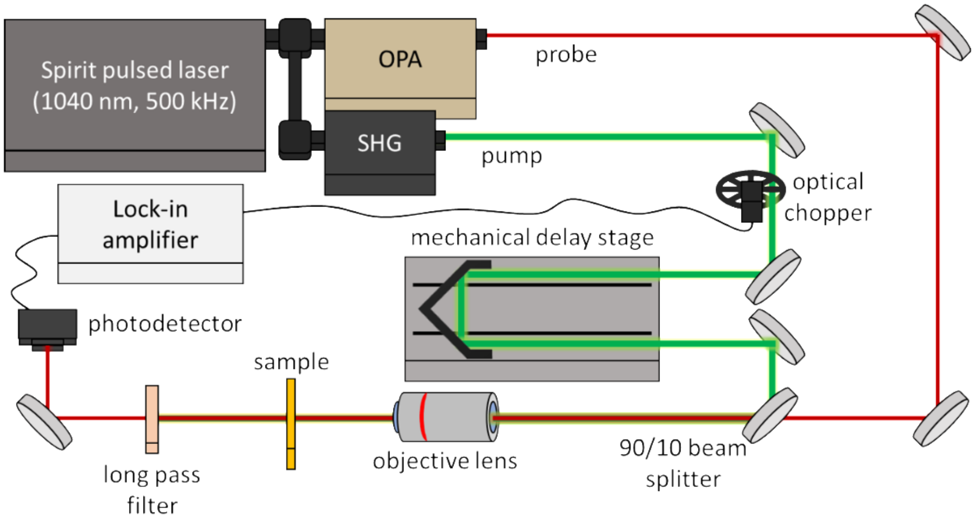

Figure S1. The experimental setup for low-perturbation pump-probe technique including the pump beam to heat the sample and a probe beam with wavelength tunability in the near IR to measure the changes in the optical properties of VO_2.

By solely considering the highest fluence, it appears that the MIT was triggered instantaneously *prior* to electron-phonon coupling. According to the two-temperature and steady-state heating models, one would expect that the lattice gains energy more rapidly at higher fluences. However, our findings indicate that the transition consistently happens at a specific threshold phonon temperature within sub-picosecond timescales. In extending this hypothesis to earlier works, we calculated the lattice temperature rise using TTM at the timescale of the reported phase transition; we find the reported MIT time is consistently associated with temperatures far in excess of the equilibrium transition temperature. In other words, in prior works, the *lattice* is calculated here to be sufficiently hot at the time of observed switching, thus supporting the posit of a lattice-mediated transition.

Supporting 2 - Computational Details:

The ab initio calculations of electronic structure, geometry optimization, and ground state molecular dynamics (MD) simulations are performed using density functional theory (DFT) with the Vienna Ab initio Simulation Package (VASP) [3-5]. The Perdew−Burke−Ernzerhof (PBE) exchange-correlation functional and the projector-augmented wave (PAW) method are employed along with DFT+U method (U=4.0, J=0.6) for V atoms[6-8]. The plane-wave energy cutoff is set as 520 eV, and 3 x 3 x 3 k-point mesh centered on the Γ-point is used to sample the Brillouin zone.

The pristine VO_2 system is represented by a 2 x 2 x 2 conventional monoclinic supercell. After geometry optimization, the other two systems containing defects are created by removing one oxygen atom (oxygen vacancy O_v) and adding one oxygen atom (oxygen interstitial O_i). Further geometry optimization is performed on these two systems to relax the strain near the defects. The three systems are then gradually heated to 300 K and relaxed for 3 ps with constant temperature using the Nose-Hoover thermostat, followed by 3 ps simulations with the microcanonical ensemble. The last 1 ps is used to compute the nonadiabatic coupling for the modeling of excited state dynamics.

The nonadiabatic MD simulations are performed using surface hopping implemented within the real-time time dependent DFT framework. The initial excitation corresponded to promotion of an electron from the valence band maximum (VBM) to the conduction band minimum (CBM). In order to achieve converged results, we considered states within several k_BT of thermal energy above the initially excited state. For this reason, the nonadiabatic MD simulations included VBM, CBM, midgap states, 5 additional bands below the VBM and 5 bands above the CBM. We use the PYthon eXtension for Ab Initio Dynamics (PYXAID) code for computing the NAC and performing the nonadiabatic MD simulations[9,10]. The decoherence-induced surface hopping (DISH) method is used with 100 initial geometries and 1000 random number sequencies for each initial geometry[11].

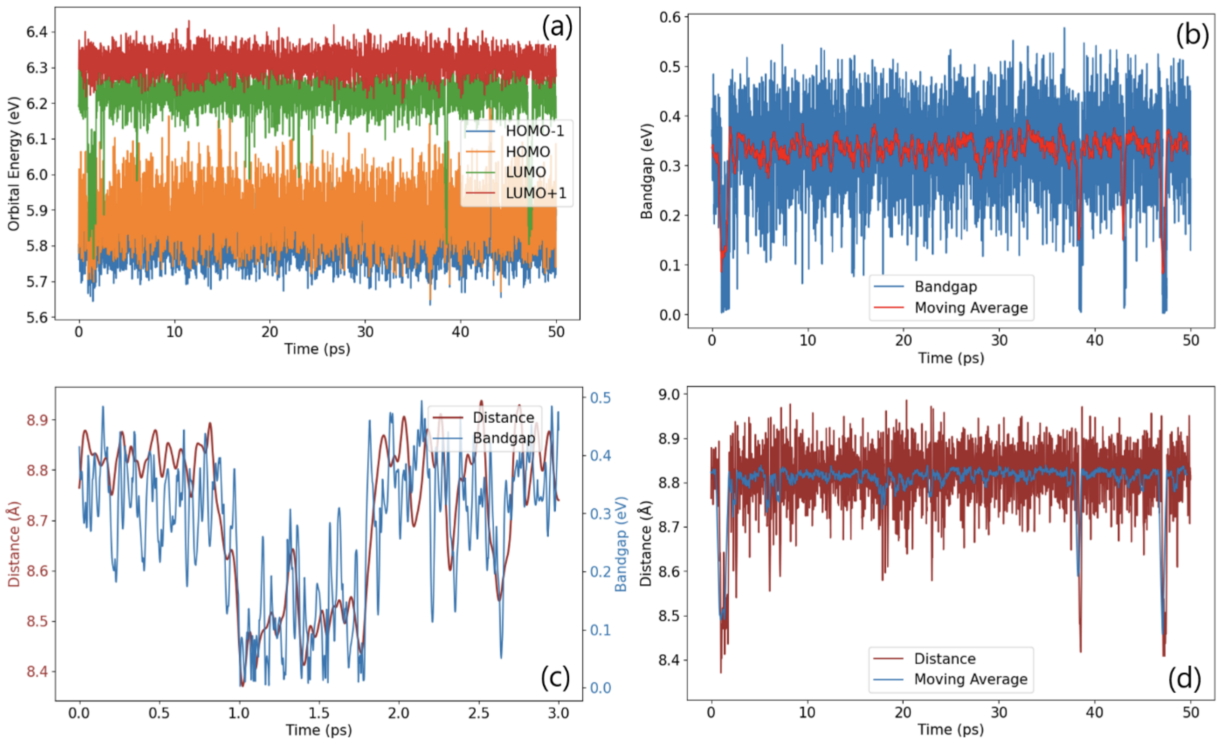

Figure S2. **(a)** Evolution of energy levels near the bandgap in VO_2 with O vacancy over 50 ps. Repeatedly during the trajectory the bandgap approaches zero, demonstrating transient metallic behavior. **(b)** Evolution of the bandgap. **(c)** Evolution of the bandgap and distance between the two V atoms highlighted in green in Figure S3b over the first 3 ps of the 50 ps trajectory. There is a strong correlation between the bandgap and the distance, exhibiting approximately a 2ps periodicity. **(d)** Evolution of the between the two V atoms highlighted in green in Figure S3b over 50 ps. There is a significant correlation between the distance and the bandgap shown above in part (b), identified by the large value of MI, Figure S3a.

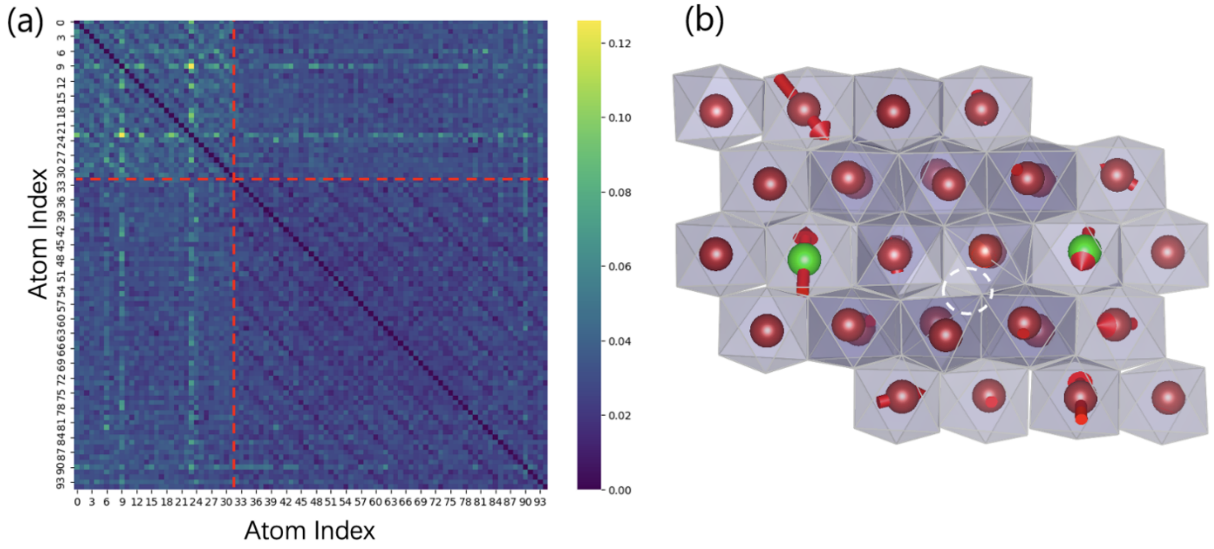

Figure S3. **(a)** Mutual information (MI) correlating the bandgap and distances between all pairs of atoms in the VO_2 system with O vacancy. The first 32 atoms are V, while the remaining 63 atoms are O. There is a strong correlation between the bandgap and distance between two V atoms shown in green in part (b), as evidenced by large MI values seen as yellow dots in top-left block. **(b)** Normal mode with 500 GHz frequency, exhibiting strong contribution from the two V atoms shown in green and having a strong correlation with the bandgap. The mode matches well with the 2 ps coherence oscillation seen in the experiment. The location of the O vacancy is shown by the white dashed circle.

Supporting References

[1] Morrison, V. R.; Chatelain, R. P.; Tiwari, K. L.; Hendaoui, A.; Bruhács, A.; Chaker, M.; Siwick, B. J. A Photoinduced Metal-like Phase of Monoclinic VO_2 Revealed by Ultrafast Electron Diffraction. *Science* **2014**, *346* (6208), 445–448.

[2] Hilton, D. J.; Prasankumar, R. P.; Fourmaux, S.; Cavalleri, A.; Brassard, D.; El Khakani, M. A.; Taylor, A. J.; Kieffer, J. C.; Averitt, R. D. Enhanced Photosusceptibility near Tc for the Light-Induced Insulator-to-Metal Phase Transition in Vanadium Dioxide. *Phys. Rev. Lett.* **2007**, *99* (22), 226401.

[3] Kohn, W. Nobel Lecture: Electronic Structure of Matter—Wave Functions and Density Functionals. *Rev. Mod. Phys.* **1999**, *71* (5), 1253–1266.

[4] Kohn, W.; Sham, L. J. Self-Consistent Equations Including Exchange and Correlation Effects. *Phys. Rev.* **1965**, *140* (4A), A1133–A1138.

[5] Hohenberg, P. Inhomogeneous Electron Gas. *Phys. Rev.* **1964**, *136* (3B), B864–B871.

[6] Blöchl, P. E.; Jepsen, O.; Andersen, O. K. Improved Tetrahedron Method for Brillouin-Zone Integrations. *Phys. Rev. B* **1994**, *49* (23), 16223–16233.

[7] Perdew, J. P.; Burke, K.; Ernzerhof, M. Generalized Gradient Approximation Made Simple. *Phys. Rev. Lett.* **1996**, *77* (18), 3865–3868.

[8] Huefner, M.; Mildenberger, D.; Schramm, S.; Chudoba, C.; Reyren, N.; Villemin, T.; Bicchi, M.; Giusti, F.; Fournel, F.; Pocholle, J. P. Hubbard Gap Modulation in Vanadium Dioxide Nanoscale Tunnel Junctions. *Nano Lett.* **2014**, *14* (11), 6115–6120.

[9] Akimov, A. V.; Prezhdo, O. V. The PYXAID Program for Non-Adiabatic Molecular Dynamics in Condensed Matter Systems. *J. Chem. Theory Comput.* **2013**, *9* (11), 4959–4972.

[10] Akimov, A. V.; Prezhdo, O. V. Advanced Capabilities of the PYXAID Program: Integration Schemes, Decoherence Effects, Multiexcitonic States, and Field-Matter Interaction. *J. Chem. Theory Comput.* **2014**, *10* (2), 789–804.
[11] Jaeger, H. M.; Fischer, S.; Prezhdo, O. V. Decoherence-Induced Surface Hopping. *J. Chem. Phys.* **2012**, *137* (22), 22A545.
