# Supplementary material for: Ultrafast Charge Carrier Dynamics in Vanadium Dioxide, VO2: Nonequilibrium Contributions to the Photoinduced Phase Transitions
Source: J Phys Chem Lett. 2025 Jan 28;16(5):1312–9. doi: 10.1021/acs.jpclett.4c02951 (PMC11808786; doi:10.1021/acs.jpclett.4c02951)
Supplement: Supplementary file 2 — jz4c02951_si_002.pdf [file jz4c02951_si_002.pdf]

jz-2024-02951q.R1

Name: Peer Review Information for "Ultrafast charge carrier dynamics in Vanadium Dioxide, VO2: Non-equilibrium contributions to the photo-induced phase transitions"

First Round of Reviewer Comments

Reviewer: 1

Comments to the Author

In the manuscript entitled "Ultrafast charge carrier dynamics in Vanadium Dioxide, VO_2: Non-equilibrium contributions to the photo-induced phase transitions" by Tomko *et al.*, the authors claimed that they investigated ultrafast dynamics of photo-induced phase transition in VO_2 under low perturbation conditions. However, the experiments are carried out in the insulating phase. The findings indicate that transient optical properties undergo significant alterations even with a minimal number of electronic excitations, which do not trigger an insulator-metal transition (IMT). With the assistance of first-principle simulations, the authors propose that the observed changes in optical properties are attributed to shifts in the Fermi level and variations in the density of states, which arise from local defects in VO_2 following pump laser irradiation.
This paper seeks to clarify the relationship between transient spectroscopy and IMT. However, there is a lack of comparative experimental data presented in the metal phase. From this perspective, I cannot recommend that the manuscript be accepted for publication in this high-impact journal.

I suggest the authors undertake substantial revisions of their manuscript and submit the revised manuscript to a specific journal, for example, Journal of Physical Chemistry C. Specifically, the authors should include direct comparisons of transient spectroscopic data from both insulating and metallic phases of VO_2 and further explore how these relate to IMT. The authors can revise the manuscript according to following comments and questions.

1. The observation of the increase in probe light reflectivity following pump laser irradiation has been reported and is attributed to a local IMT phase transition (Opt. Mater. Express 10, 1393-1404 (2020)), which is not cited in the manuscript. The observation is very similar to the present manuscript. However, the authors explained the changes of optical properties using Fermi level shifts and density of state variations caused by local defects in VO_2 following pump laser irradiation. Could the authors analyze the different mechanism responsible for the similar change of transient optical properties after pump lase irradiation?

2. What is the substrate? The thermal electron transfer at the interface can have a nonnegligible impact on the material properties for nanofilm samples. Can the authors rule out the effect of the substrate?
3. Is there any damage for sample after pump laser irradiation? The authors claimed that the changes of optical properties are caused by local defects in VO_2 after pump laser irradiation. Is the process reversible?
4. The authors found that the changes of the optical properties (reflectivity) of VO_2 have been taken place before IMT. The authors should carry out experiments to measure the changes of the optical properties after IMT and compare the changes of optical properties before and after IMT?
5. In the present high-repetition laser rate measurements, the 'temperature rises' is attributed to the accumulation of energy between multiple pump pulses and the single-pulse excitation of the system. In fact, these two contributions can be separated by measuring the probe laser signal with and without the pump laser during scanning the time delay between the two laser pulses.
6. The presentation should be reviewed and enhanced to improve its readability. For example: The "literature results" presented in Fig. 1(d) should provide proper citation of the data sources. The highly-periodic oscillation in Fig. 2(d) should be explained in the main text.

Reviewer: 2

Comments to the Author

In this work, the photo-induced phase transformation of VO2 was investigated. The authors adopted an approach that provides a steady-state temperature slightly below the MIT temperature. Therefore, only a small T_pulsed is needed to exceed the phase transition temperature of VO2, allowing low energy optical pulses without inducing strong nonlinearities. A re-rise in the transient reflectance was observed in this low perturbation condition, but further analysis suggests that the re-rise is not associated with the first-order phase transformation. Instead, the shift of DOS and Fermi level due to nuclear motion of oxygen defects is responsible. Theoretical calculations were also performed to support this mechanism. The results are quite interesting and the findings provide new insights into the origin phase transformation of VO2. I recommend the publication of this work after the following issues are addressed.

1. It'll be good to specify how many bands were included in the NAMD simulations. Would the results strongly depend on the number of bands?

2. Since PYXAID adopts the CPA, the nuclear trajectory is generated by the normal ground-state BOMD. Thus the band dynamics in Fig. 3b, e, h do not include effects from the excited electrons. According to these figures the system is already somewhat metallic at 300 K when oxygen defects exists. Hence it is not very clear to me how the optical excitation enhances the metallic character of

VO2. There could be two scenarios: (i) When the temperature increases from 300 K to slightly above the transition temperature (say 400 K), the motion of oxygen defects greatly enhances so the DOS shift is significantly larger; (ii) The optical excitation significantly increases the concentration of oxygen defects. Which one is more likely to occur in reality? Or could there be other possibilities?

Author's Response to Peer Review Comments:

We greatly appreciate the Reviewer's time and effort in reviewing our manuscript. Below we copy each Reviewer's comments **in bold** and our responses and revisions in blue.

Reviewer 1

In the manuscript entitled "Ultrafast charge carrier dynamics in Vanadium Dioxide, VO2: Nonequilibrium contributions to the photo-induced phase transitions" by Tomko et al., the authors claimed that they investigated ultrafast dynamics of photo-induced phase transition in VO2 under low perturbation conditions. However, the experiments are carried out in the insulating phase. The findings indicate that transient optical properties undergo significant alterations even with a minimal number of electronic excitations, which do not trigger an insulator-metal transition (IMT). With the assistance of first-principle simulations, the authors propose that the observed changes in optical properties are attributed to shifts in the Fermi level and variations in the density of states, which arise from local defects in VO2 following pump laser irradiation. This paper seeks to clarify the relationship between transient spectroscopy and IMT. However, there is a lack of comparative experimental data presented in the metal phase. From this perspective, I cannot recommend that the manuscript be accepted for publication in this high-impact journal.

I suggest the authors undertake substantial revisions of their manuscript and submit the revised manuscript to a specific journal, for example, Journal of Physical Chemistry C. Specifically, the authors should include direct comparisons of transient spectroscopic data from both insulating and metallic phases of VO2 and further explore how these relate to IMT. The authors can revise the manuscript according to following comments and questions.

Authors' response: We would like to thank the reviewer for taking the time to review our manuscript and their feedback. In the following, we provide a point-by-point response to all the comments raised by the reviewer.

1. The observation of the increase in probe light reflectivity following pump laser irradiation has been reported and is attributed to a local IMT phase transition (Opt. Mater. Express 10, 1393-1404 (2020)), which is not cited in the manuscript. The observation is very similar to the present manuscript. However, the authors explained the changes of optical properties using Fermi level shifts and density of state variations caused by local defects in VO2 following pump laser irradiation. Could the authors analyze the different mechanism responsible for the similar change of transient optical properties after pump lase irradiation?

Authors' Response: We greatly appreciate the comparison to this local IMT phase transition. Indeed, this work observes qualitatively comparable observations in their pump-probe response, *particularly at thermal timescales* (100s of ps to nanoseconds). However, the critical difference is on the excitation and probing methodologies, which reflect the strong differences in data. These differences in data are what provides unique insight via the submitted article.

In the referenced paper (Opt. Mater. Express 10, 1393-1404 (2020)), the observed fluences for the IMT are nearly one order of magnitude lower than observed in this work, despite our use of a high-repetition rate oscillator. This is mostly attributable to the fact that they are not directly exciting the VO2, but, rather, most of the pump energy is absorbed in the semiconductor substrate (TiO2 and doped TiO2). They are thus exploring the energy transfer from the substrate into the VO2 film and its effect on the IMT. In contrast, this work utilizes a non-absorbing substrate that provides negligible differential optical response (i.e., dR/R) during these experiments, allowing direct insight to the VO2's, and only the VO2's, optical behavior.

This difference of excitation methods leads to a long-duration, thermal-like response that provides insight to energy transfer from the substrate into the overlying film. Our research group has studied this phenomenon in extreme depth in multiple material systems (see *Nature Nanotechnology* **16**, 47-51 (2021), *Applied Physics Letters* **112**, 171602 (2018), *ACS Applied Materials & Interfaces* **9**, 43343 (2017)., J*ournal of Applied Physics* **117**, 044305 (2015)).

Most importantly, our work *does* show similar behavior in that the pump-pulse does directly induce the IMT once the IMT threshold temperature is reached. In our work, however, this IMT is found to occur following electron-phonon equilibration occurs within the film, an aspect that cannot be observed in the referenced article due to these differences in experimental conditions. This finding is impactful to the studies of IMTs in general, particularly in VO2, as multiple referenced articles argue hot-electron driven transitions, whereas our work indisputably shows this is not the case. The oxygen defects, on the other hand, are what give rise to the observed oscillations, which are again only observable through direct detection/excitation of the thin VO2 film without obfuscation from the underlying substrate.

2. What is the substrate? The thermal electron transfer at the interface can have a non-negligible impact on the material properties for nanofilm samples. Can the authors rule out the effect of the substrate?

Authors Response: We appreciate the reviewer's concern regarding the electrons contribution to the thermal transport at the interface. The substrate is Sapphire which is a dielectric. We updated the figure in the manuscript to better reflect this:

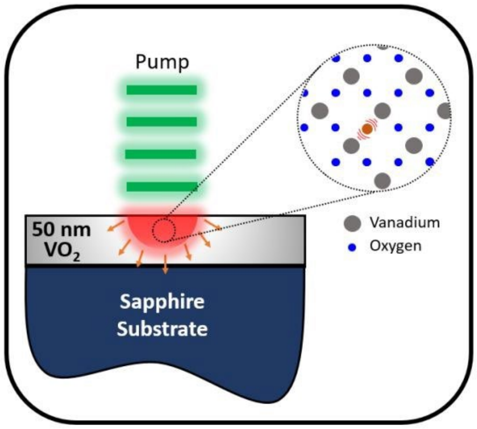

Electrons are generally the most efficient heat carriers at metal/metal interfaces. However, at metal/nonmetal interfaces, the electron-phonon coupling factor determines whether a portion of heat is transferred from electrons to the substrate. For metal/non-metal interfaces, heat propagation occurs through two primary pathways: (i) phonon-phonon and (ii) electron-phonon interactions (as illustrated in [1]).

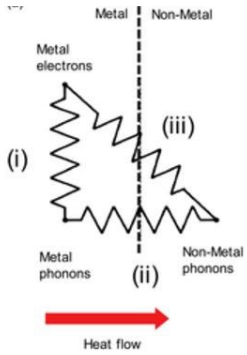

Differentiating between these two mechanisms is a complex task and beyond the scope of this study. Regardless, electron-driven thermal transport—whether negligible or not—affects interfacial thermal transport and contributes to the resulting temperature rise in the thin film. Importantly, we do not expect significant changes in material properties, apart from minor temperature variations. Since precise temperature knowledge is not critical to our study, the results are not expected to be influenced by the electronic contribution at the interface.

References:

[1] Giri, Ashutosh, and Patrick E. Hopkins. "A review of experimental and computational advances in thermal boundary conductance and nanoscale thermal transport across solid interfaces." Advanced Functional Materials 30.8 (2020): 1903857.

3. Is there any damage for sample after pump laser irradiation? The authors claimed that the changes of optical properties are caused by local defects in VO2 after pump laser irradiation. Is the process reversible?

Authors Response: There is no damage to the sample following pulsed laser irradiation. The local defects are indeed reversible under these experimental conditions. This reversibility is “built-in” to the experimental method used here: our pump-probe systems in this work operate at >1 MHz repetition rates, where each data point is generally acquired for multiple seconds, such that each data point within the observed oscillation is the average signal produced by >2 million laser pulses. Then, the pump-probe delay time is changed, and the next >2 million laser pulses occur, producing the next signal. For the oscillations to arise as a function of pump-probe delay time indicates that these 2 million laser pulses do not induce permanent changes.

This is also supported in that the same sample is irradiated at high and low intensities, repeatedly, to ensure self-consistent measurements can occur (i.e., we repeat our measurements to check for laserinduced damage).

4. The authors found that the changes of the optical properties (reflectivity) of VO2 have been taken place before IMT. The authors should carry out experiments to measure the changes of the optical properties after IMT and compare the changes of optical properties before and after IMT?

Authors Response: The reviewer brings up an interesting aspect of pump-probe measurements that is critical toward interpretation of the results presented here. It is important to remember that the majority of these data are displaying the *differential* reflectivity, or change in optical properties, due to pump-pulse excitation. In systems lacking an IMT (e.g., gold films), there will still be an observed change to the optical properties (pump probe signal) because the sensitivity of the system can resolve temperature-driven changes in reflectivity on the order of <<1% changes to optical properties.

The observed changes to the optical properties, i.e., the differential reflectivity, prior to the IMT are simply the result of transient heating of the sample. Then, when the IMT occurs, the differential reflectivity is much greater and displays different transient behavior.

We observe the *baseline* optical properties as a metric of potential damage or non-reversible changes to the optical properties of the film (i.e., we produce dR/R, where R is the “baseline” optical properties, by measuring dR and R independently). Indeed, R is found to be unchanged over the course of these experiments, suggesting that there is no change in optical reflectivity before and after the *transient* IMT occurs. Of course, *during* the IMT, there are extreme changes in optical properties as shown here!

5. In the present high-repetition laser rate measurements, the ‘temperature rises’ is attributed to the accumulation of energy between multiple pump pulses and the single-pulse excitation of the system. In fact, these two contributions can be separated by measuring the probe laser signal with and without the pump laser during scanning the time delay between the two laser pulses.

Authors Response: The reviewer brings up an interesting method to measuring the non-instantaneous contribution to the reflectivity. Indeed, the two contributions *are* measured

independently in our experimental setup, but through an alternate method. Because we utilize a lock-in amplifier that is tuned to the modulation frequency of the pump pulse, we can technically measure the in-phase (instantaneous) and out-of-phase ("accumulated") response of reflectivity changes during our experiments without needing to vary the experimental apparatus as suggested here (i.e., the reviewer's suggestion of scanning the probe laser signal with no pump is interesting, but if the pump is off, then there's neither instantaneous single pulse responses nor accumulated response – we rely on the pump pulse being present for *all* measurements).

Indeed, the majority of the transient behavior presented in this work is in-fact due to the in-phase, instantaneous contribution to the thermal signal. The main advantage to the baseline "accumulated" signal is that each individual pulse can operate at comparatively low (~order of magnitude less than literature) fluences; the accumulated pulse essentially does nothing more than act as a well-calibrated furnace. In doing so, the sample can be "background" heated to 339 K, then a single low-fluence pulse can induce a temperature rise of 1-2 K, causing the lattice temperature to exceed 340 K. Indeed, once the lattice temperature reaches 340 K, a phase transition is observed – this response is dominated by the in-phase, instantaneous signal.

The critical component on this aspect, relevant to our work, is that there is a temporal lag between the excitation pulse arriving at the sample, which heats the electrons in the material slightly, and the lattice beginning to heat-up due to slow electron-phonon coupling under these low fluences. Once the electrons lose energy to the lattice and 340 K is reached, the phase transition is observed. However, when the excitation pulse does not cause the sample to exceed 340 K, no phase transition is observed.

6. The presentation should be reviewed and enhanced to improve its readability. For example: The "literature results" presented in Fig. 1(d) should provide proper citation of the data sources. The highly-periodic oscillation in Fig. 2(d) should be explained in the main text.

Authors Response: Thank you for pointing to this omission in our manuscript. All the necessary references related to Fig. 1(d) are added to the main manuscript, in the caption. Additionally, the discussion related to Fig. 2(d) is addressed later in the manuscript, on the last line of page 6, where the computational results are discussed as follows:

"As shown in Fig.2d, our ultrafast pump-probe data displays oscillatory behavior with a periodicity of approximately 2 picoseconds; this period is far too-long to be associated with the phonon dynamics of VO2, picosecond acoustics in the film itself, or Brillouin scattering within the sapphire substrate. However, this period is on the order of the fluctuation of the energy level that crosses the band gap in the oxygen vacancy system observed in our TD-DFT calculations, Figure 3e."

Reviewer 2

In this work, the photo-induced phase transformation of VO2 was investigated. The authors adopted an approach that provides a steady-state temperature slightly below the MIT temperature. Therefore, only a small T_pulsed is needed to exceed the phase transition temperature of VO2, allowing low energy optical pulses without inducing strong nonlinearities. A re-rise in the transient reflectance was observed in this low perturbation condition, but further analysis suggests that the re-rise is not associated with the firstorder phase transformation. Instead, the shift of DOS and Fermi level due to nuclear motion of oxygen defects is responsible. Theoretical calculations were also performed to support this mechanism. The results are quite interesting and the findings provide new insights into the origin phase transformation of VO2. I recommend the publication of this work after the following issues are addressed.

Authors Response: We are grateful to the Reviewer for spending the time on the manuscript, for its positive evaluation and the constructive suggestions below.

1. It'll be good to specify how many bands were included in the NAMD simulations. Would the results strongly depend on the number of bands?

Authors Response: In order to achieve converged results, one needs to consider states within several kT above the initially excited state, corresponding to excitation of an electron from the VBM to the CBM. For this reason, we included VBM, CBM, midgap states, and 5 additional bands below the VBM and 5 bands above the CBM. We have provided this information in the Computational Details section of Supporting Information during the revision.

2. Since PYXAID adopts the CPA, the nuclear trajectory is generated by the normal ground-state BOMD. Thus the band dynamics in Fig. 3b, e, h do not include effects from the excited electrons. According to these figures the system is already somewhat metallic at 300 K when oxygen defects exists. Hence it is not very clear to me how the optical excitation enhances the metallic character of VO2. There could be two scenarios: (i) When the temperature increases from 300 K to slightly above the transition temperature (say 400 K), the motion of oxygen defects greatly enhances so the DOS shift is significantly larger; (ii) The optical excitation significantly increases the concentration of oxygen defects. Which one is more likely to occur in reality? Or could there be other possibilities?

Authors Response: This is a great point to consider, thank you. Indeed, the current calculations are performed under the CPA using the ground state BOMD trajectory. The simulations show that in the presence of oxygen vacancies, the system exhibits large fluctuations of the energy levels across the bandgap already at ambient conditions. The simulations also show that photoexcited electrons in this system rapidly (3ps) relax across the bandgap, depositing the excess electronic energy into vibrations. This increases the local temperature of vibrations around the oxygen vacancy defects, thereby enhancing the thermal fluctuations of electronic energy levels. Thus, the simulations support mechanism (i) suggested by the Reviewer. We explicitly discuss this point in the revised manuscript. Please, see the paragraph below Figure 3.

Regarding the second proposal by the Referee: it is feasibly that long time irradiation of the system can create additional structural damage and increase defect concentration. This process can occur on a much longer timescale than the simulation or even experimental time of the current study.

jz-2024-02951q.R2

Name: Peer Review Information for "Ultrafast charge carrier dynamics in Vanadium Dioxide, VO2: Non-equilibrium contributions to the photo-induced phase transitions"

Second Round of Reviewer Comments

Reviewer: 1

Comments to the Author

The authors have partially addressed the questions and comments raised by the referees. They assert that their optical pump-probe experiments align excellently with prior literature, where a 're-rise' in transient reflectance has been attributed to the MIT of VO2. This conclusion is crucial for this manuscript. However, the authors do not provide detailed references or clarify why the 're-rise' in transient reflectance can be ascribed to the MIT. Recent experiments on phase transitions of VO2 indicate that phase identification is achieved by controlling the sample's temperature [Phys. Rev. Lett. 132, 186903 (2024), Photonics Res. 12, 2831 (2024)]. I believe that the authors have not provided solid evidences supporting their claim that a 're-rise' in transient reflectance corresponds to the MIT of VO2. Therefore, I cannot recommend acceptance of this manuscript in its current form for publication in this high-impact journal.

Author's Response to Peer Review Comments:

We greatly appreciate the Reviewer's time and effort in reviewing our revised manuscript. Below we copy each Reviewer's comments **in bold** and our responses and revisions in blue.

Reviewer 1

The authors have partially addressed the questions and comments raised by the referees. They assert that their optical pump-probe experiments align excellently with prior literature, where a 're-rise' in transient reflectance has been attributed to the MIT of VO2. This conclusion is crucial for this manuscript. However, the authors do not provide detailed references or clarify why the 're-rise' in transient reflectance can be ascribed to the MIT. Recent experiments on phase transitions of VO2 indicate that phase

identification is achieved by controlling the sample's temperature [Phys. Rev. Lett. 132, 186903 (2024), Photonics Res. 12, 2831 (2024)]. I believe that the authors have not provided solid evidences supporting their claim that a ‘re-rise’ in transient reflectance corresponds to the MIT of VO2. Therefore, I cannot recommend acceptance of this manuscript in its current form for publication in this high-impact journal.

Authors’ response: We agree that the observation of the re-rise trend in our transient thermoreflectance signal is a key to identify the MIT in VO2, and indeed in the text of manuscript we have cited several papers that have observed this, as stated on page 4 above Eq. 1:

“With increasing fluence (e.g., a fluence of ~2.5 Jm^{-2}), this secondary re-rise becomes significant, beginning at a pulse-width-limited time scale of 400 femtoseconds, and the transient reflectance increases by nearly 300%. This observation is again in agreement with prior works and has been associated to the photo-induced phase transition of VO_2.[23, 32, 33, 43, 44]” For the reviewer’s and editor’s ease in assessment, these references from our manuscript are listed again below. While these references were included in the text of the manuscript, they were not clearly listed in the caption of Fig. 1 where we also refer to this trend in transient thermoreflectance signal as being in line with previous literature. We have now added this to the caption of Fig. 1, shown below.

We also note that one of the references that the reviewer cites in their comment (PRL 132 186903) is in strong support of the findings in our paper and indeed in their experiments they observe a similar “rerise” in their pump-probe SNOM data consistent with our results and the references that we cite in our manuscript. Their conclusions that the observed re-rise is driven by the “nucleation and growth of the metallic phase” is also directly in support of the findings that we report in our paper. Thus, we have also added this PRL reference to our paper in the discussions referenced above and we thank the reviewer for notifying us of this additional paper to cite (now reference 48).

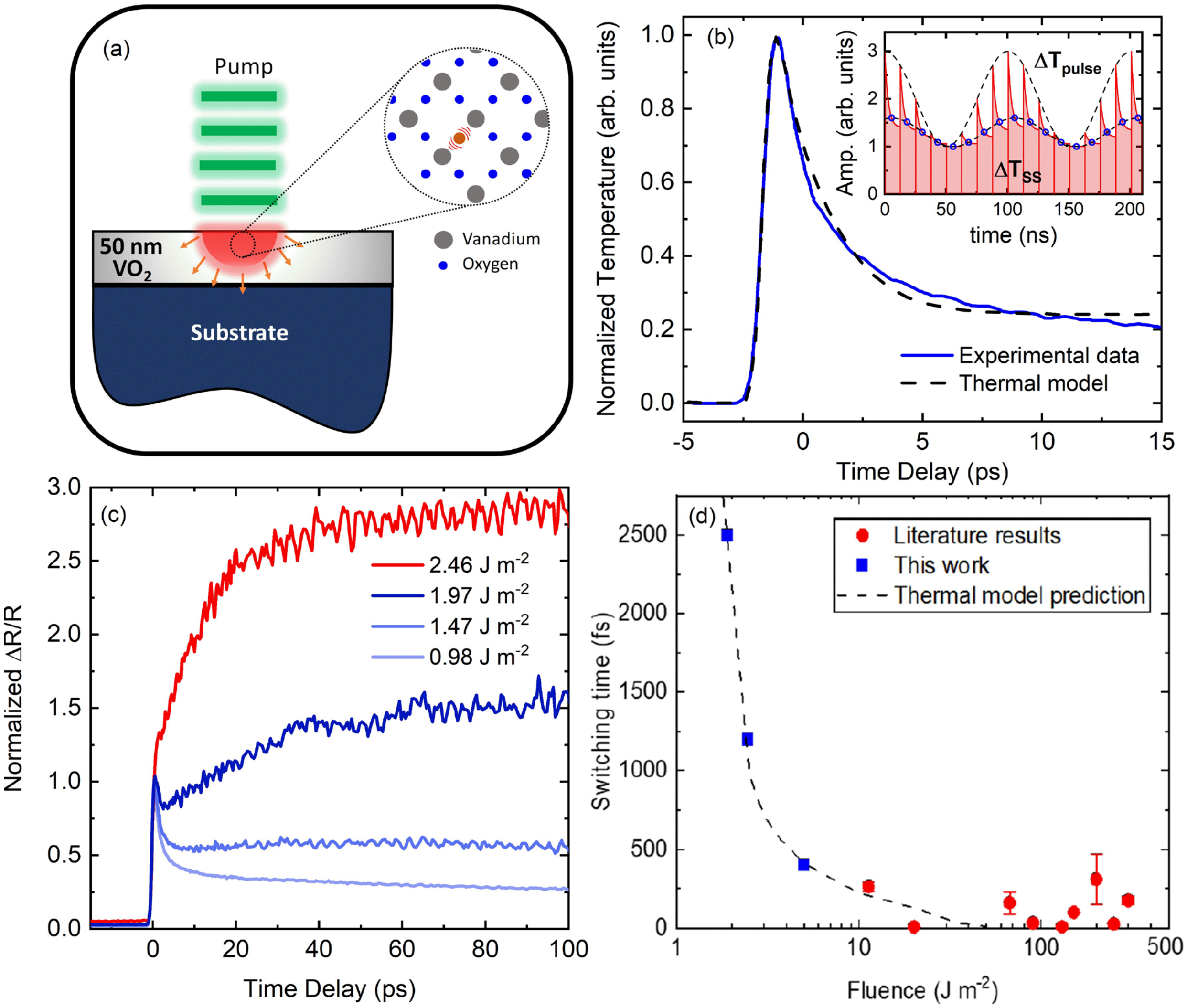

Figure 1. a) Schematic of measurements technique and the VO_2 with defects. b) Two-temperature model (TTM) fit (dashed black line) to our experimental transient reflectivity measurements (blue line), providing a detailed understanding of the energy transfer rates between electrons and phonons within VO_2. The inset shows the periodic temperature rise due to ultrafast pump-probe experiments and their corresponding exponential thermal decay. At high modulation frequencies, as used in this work, there are two contributions; a relatively constant 'background' steady-state temperature rise (!T_{SS}) due to pulse accumulation, and the more often considered 'impulse' temperature rise due to individual pulses (!T_{pulsed}). c) Transient reflectivity data measured at 800 nm for varying pump fluences. There is a marked change in dynamics beginning at 1.5 J m$\rightarrow^{2}$; this change has been previously associated with a photo-induced phase transition[23,32,33,43–45]. d) Predicted switching time for a lattice-driven phase transition (i.e., how long before the electrons couple sufficient energy to the lattice to exceed 340 K), compared to our experimental results and a number of reported transition times reported in literature[21,32,34,43,46–48].

References:

23. Donges, S. A. et al. Ultrafast nanoimaging of the photoinduced phase transition dynamics in VO_2. Nano letters 16, 3029–3035 (2016).

32. Morrison, V. R. et al. A photoinduced metal-like phase of monoclinic VO_2 revealed by ultrafast electron diffraction. Science 346, 445–448 (2014).
33. Pashkin, A. et al. Ultrafast insulator-metal phase transition in VO_2 studied by multiterahertz spectroscopy. Phys. Rev. B 83, 195120 (2011).
43. Jager, M. F. et al. Tracking the insulator-to-metal phase transition in VO_2 with few-femtosecond extreme UV transient absorption spectroscopy. Proc. Natl. Acad. Sci. 114, 9558–9563 (2017).
44. Radue, E. et al. Substrate-induced microstructure effects on the dynamics of the photo-induced metal–insulator transition in VO_2 thin films. J. Opt. 17, 025503 (2015).
45. Sternbach, A. J. et al. Inhomogeneous photosusceptibility of VO_2 films at the nanoscale. Phys. Rev. Lett. 132, 186903 (2024).
